# A quality improvement project on reimaging for provision of extended window mechanical thrombectomy when 24/7 service is not available

**DOI:** 10.1016/j.clinme.2025.100521

**Published:** 2025-10-09

**Authors:** Shadi M Ramadan, Bernard Esisi

**Affiliations:** Hull University Teaching Hospitals NHS Trust, Hull, UK

**Keywords:** Out of hours mechanical thrombectomy, Late window mechanical thrombectomy, Large vessel occlusion (LVO) stroke

## Abstract

**Background:**

Mechanical thrombectomy (MT) has revolutionised the treatment of ischaemic stroke, leading to decreased rates of disability and mortality. However, many centres are unable to deliver a 24/7 service. Consequently, many patients who present in the non-operating hours are not considered for this treatment.

**Methods:**

We conducted a quality improvement project (QIP) to provide patients presenting out of hours with access to MT. To achieve this, we designed a protocol to rescan those patients just before opening of the service to select the candidates based on the favourable perfusion criteria.

**Results:**

Twenty-two out of the 39 patients included in the QIP had MT, which was not accessible before initiation of our protocol.

**Conclusion:**

In stroke centres where 24/7 MT service is not available, patients with large vessel occlusion (LVO) stroke, who present in the non-operating hours, can get access to this treatment by consideration of early morning rescanning just before opening of the service.

## Introduction

In the UK, it is estimated that 10% of stroke patients are eligible for mechanical thrombectomy (MT).^1^ However, only 3.3% of the eligible patients access this treatment, compared to 5–10% of patients in northern and central Europe.^2^ A 2021 survey of 23 active MT centres in the UK showed that only four centres were providing a 24/7 service and three of them are in London.^3^

The MT service at Hull Royal Infirmary started in 2017 to cover North and East Yorkshire, and North Lincolnshire. This service is only available from 8:00 am to 4:00 pm, Monday to Friday, leaving the remainder of the working day and weekends uncovered. From November 2022, we managed to increase our operating hours until 8:00 pm on weekdays; however, this extended service was terminated in March 2024, due to staffing issues.

To address the issue of out of hours MT, we did an audit on patients who presented out of hours from January 2021 to March 2022. We found that only 17% of those patients received MT. Selection of those patients was favoured by their early-morning presentation shortly before the opening of the MT service at 8:00 am. In view of these results, we designed a protocol to consider repeating imaging of the patients who present out of hours for reconsideration for MT the next morning when the service becomes available.^4^

The details of this audit were published in the clinical medicine journal:

*A retrospective audit of out-of-hours mechanical thrombectomy of anterior circulation large vessel occlusion in a UK tertiary centre – PubMed*.^5^

The aim of our project is to provide patients presenting out of hours with access to MT based on the favourable mismatch imaging criteria, defined as mismatch volume <70 mL, mismatch ratio >1.8 and penumbra of ≥15 mL.^6^ To achieve this goal, we commenced a protocol of repeating CT head, CT angiogram and perfusion scan early in the morning for eligible patients starting from August 2022. This protocol was agreed upon between stroke physicians, neuroradiologists and interventional radiologists and was also generalised to the district hospitals in North Yorkshire and the Humber region, from where we receive referrals for MT.

## Methods

### Eligibility

Inclusion criteria (all these criteria must apply):

• Patients presented between 4:00 pm and 8:00 am Monday to Thursday (MT available after 8:00 am the next working day).
• Patients presented to hospital at any time on a Sunday (MT would be available the following Monday).
• Onset of symptoms was <24 h from the next available time for MT.
• Premorbid pre-stroke modified Rankin score (mRS) ≤3.
• National Institutes of Health Stroke Scale (NIHSS) ≥6.
• Evidence of anterior circulation large vessel occlusion (LVO) on imaging.

### Exclusion criteria

Premorbid mRS >3.
NIHSS <6.
Post-lysis NIHSS <6 (if the patient had been thrombolysed).
No evidence of LVO on imaging/low mismatch on perfusion.
Onset of symptoms >24 h from the next available time for MT.
Patients presented on Saturday or after 4:00 pm on Friday (because MT service would not be available within the following 24 h).

### Data collection

We reviewed 81 patients who presented out of hours (from 4:00 pmto 8:00 am) with ischaemic stroke due to anterior circulation LVO from August 2022 to the end of June 2024. We excluded 14 patients because they presented when the extended thrombectomy service was available (from 4:00 pm to 8:00 pm). Also, we excluded 15 patients because they did not meet the eligibility criteria. Learning from our baseline audit, we did not include 11 patients who presented in the early morning hours (0–4 h from opening of the service) because those patients would not need repeated imaging and would undergo the procedure when the service opens at 8:00 am (see Table 1). The remaining 39 patients were included in the quality improvement project (QIP).

**Table 1:** Depicts the patients who were excluded from the final analysis.

Cause of exclusion	Number of patients
Presentation within the extended MT service (from November 2022 to March 2024)	14
Presentation in the early morning hours	11
NIHSS on presentation <6	6
Post-lysis NIHSS <6	3
Posterior circulation stroke	3
Premorbid mRS >3	3
Frailty	1
Unfavourable perfusion criteria on the first scan	1

### Measures

*Process measure:* The proportion of eligible patients who will have repeated imaging in the morning when the MT service becomes available.

*Outcome measure:* The proportion of the patients presenting out of hours who will have access to MT based on the favourable imaging criteria.

*Balancing measure:* The proportion of the patients who will have repeated imaging but will not have the procedure. Those patients will have an extra dose of radiation and contrast, but will not have the procedure.

### Analysis

Data were analysed retrospectively on a 4-monthly basis from August 2022 to June 2024 using the SSNAP record at Hull Royal Infirmary (Table 2).

**Table 2:** Describes the baseline characteristics of the patients included in the QIP.

Category	Value
**Total patients**	39
**Gender**	
Men	24 (67%)
Women	15 (33%)
**Mean age**	70 years
**Patients ≥80 years**	12 (31%)
**Source**	
Comprehensive stroke centre	33 patients admitted directly to Hull Royal Infirmary
District hospitals	6 patients referred from York and Scunthorpe Hospitals
**Thrombolysis**	
Yes	26 patients
No	13 patients
**Initial NIHSS score**	
6–12	9 patients (23%)
13–20	20 patients (51%)
>20	10 patients (26%)
**Median NIHSS**	
Before thrombolysis	18
After thrombolysis	10

## Results

### Patients underwent repeat imaging

**Fig. 1:**
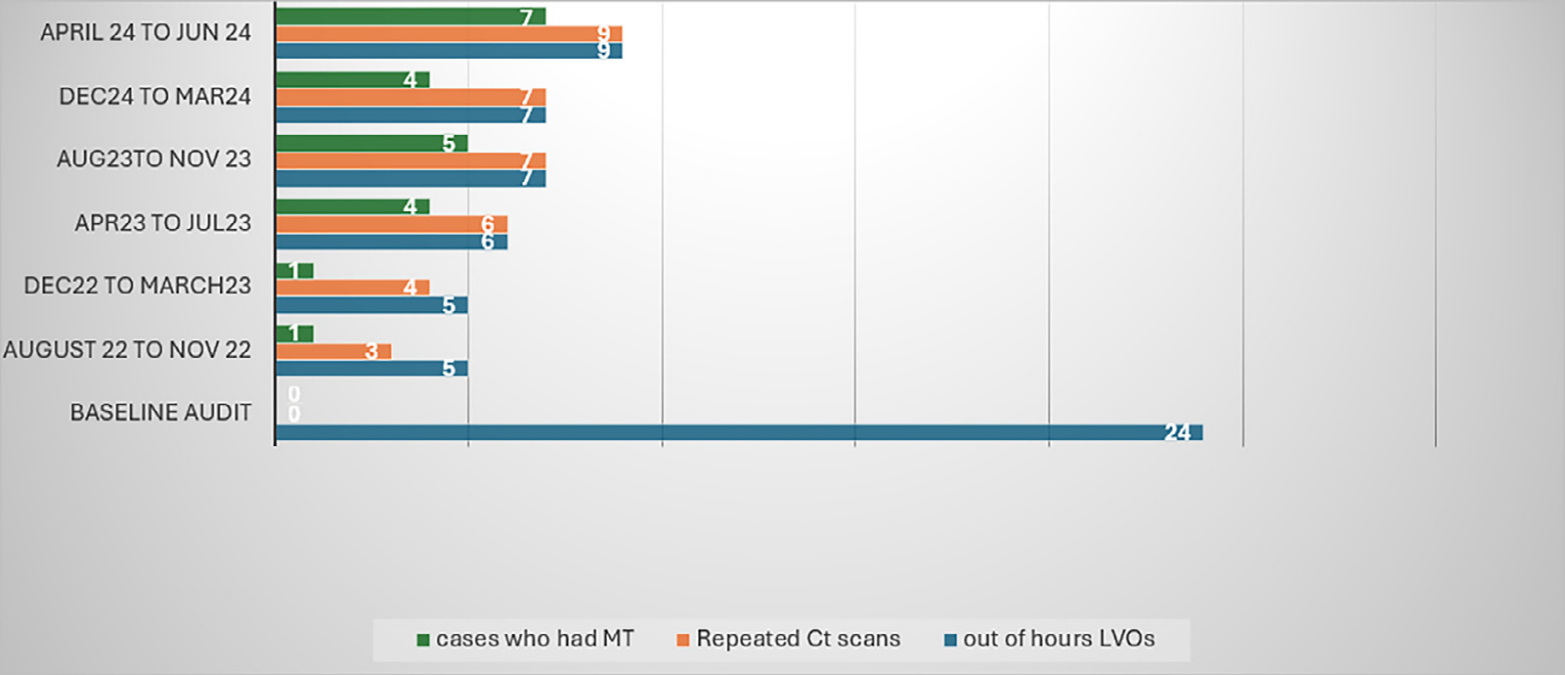
A chart depicting the 4-monthly results of the QIP from August 2022 to June 2024 compared to the baseline audit. The blue column represents the total number of patients who presented out of hours with LVO. The orange column represents the number of patients who had a repeated scan. The green column represents the number of patients who underwent MT.

Thirty-six patients, representing 92% of the included patients, were considered for repeated imaging in the morning (see Fig. 1). The three patients who were not considered for reimaging presented at the beginning of the project from August 2022 till December 2022. Since January 2023, all patients (100%) were considered for repeated imaging.

### Patients underwent thrombectomy

**Fig. 2:**
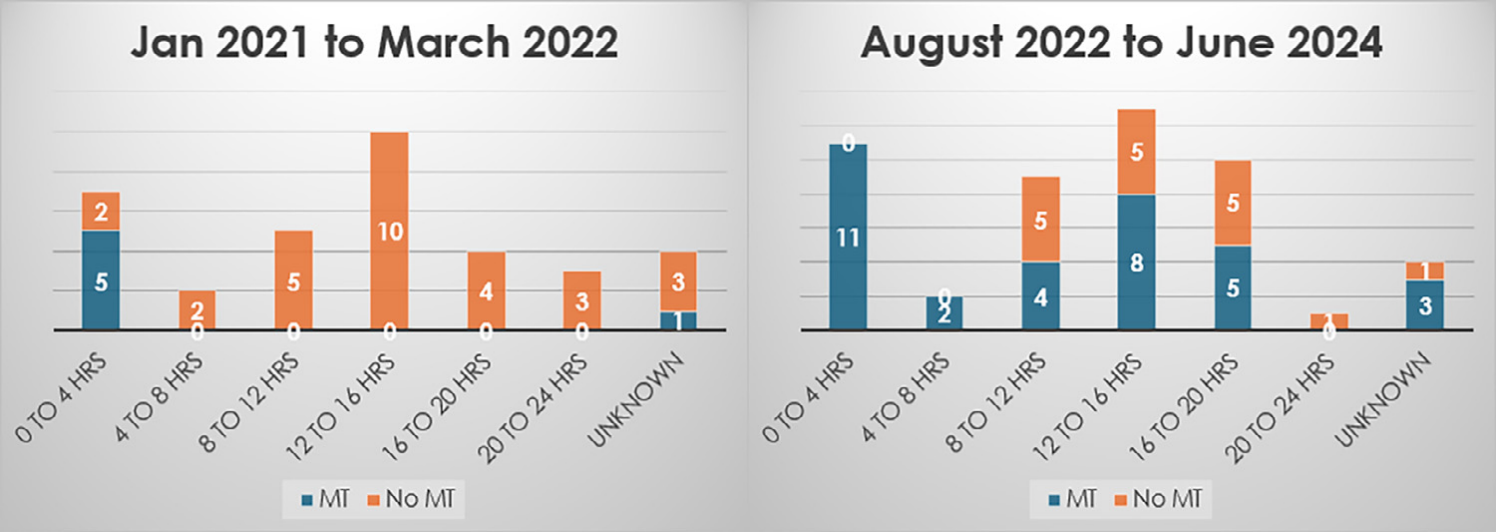
Comparison between number of patients who managed to have MT before and after the QIP based on the time of stroke onset to the opening time of MT service.

Twenty-two patients, representing 56% of the 39 patients included in this QIP, had undergone MT compared to 0% before the introduction of our protocol (see Fig. 1). In contrast to the baseline audit, this wide range of patients were treated using our protocol, irrespective of the time interval from symptom onset if it happened within the 24-h window for MT (Fig. 2).

### Patients not considered for thrombectomy

Fourteen patients (representing 38%) out of the 36 patients who had repeated scans in the morning were not considered for MT. The reasons for this are summarised in Table 3.

**Table 3:** Depicts the results of the balancing measure.

Reason why a patient was not considered for MT	Number
Reduced mismatch volume in the repeated perfusion scan	5
Re-canalisation was achieved in the repeated CT angiogram scan after thrombolysis	7
Discretion of the treating team (those two patients’ ages were 87 and 89 years and were considered too frail to have the procedure by the interventional radiologist).	2

### Functional outcome

The mRS on discharge was not documented in the notes of six patients. The mRS on discharge of the remaining 33 patients is summarised in Table 4. It is worth mentioning that DEFUSE-3 has an inclusion baseline mRS of 0–2. However, three patients with mRS of 3 were considered for MT based on the discretion of the treating physician (two patients presented when the extended service was available and one patient had MT after repeated scan in the morning. All of those three patients died because of stroke (mRS of 6).

**Table 4:** Modified Rankin score (mRS) at discharge of the patients included in the QIP.

MRS	Patients who had a repeat imaging and MT	Patients who had a repeat imaging without MT	Patients who did not have a repeat imaging
0	0	4	–
1	5	3	–
2	4	–	–
3	5	1	1
4	3	1	1
5	1	1	–
6	2	–	1

## Discussion

The benefit of MT on the prognosis of ischaemic stroke due to LVO has been established in many clinical trials. For example, HERMES metanalysis^7^ showed that MT within 12 h from stroke onset improved the absolute rate of functional independence by 20%. The number needed to treat (NNT) to reduce disability by one level of mRS was 2.6. The AURORA study^8^ enrolled patients who had MT 6–24 h from stroke onset, from six randomised trials including DEFUSE-3^5^ and DAWN trials.^9^ The NNT to achieve functional independence in AURORA meta-analysis was four. Even patients with large core infarction can still benefit from MT, as evidenced by the RESCUE-Japan^10^ trial and ANGEL-ASPECT trial.^11^

Most of the comprehensive stroke centres in the UK are unable to provide a 24/7 MT service due to logistic barriers such as inadequate staffing and lack of capital funds. The hospital cost of extending the MT service to cover 24/7 is estimated to be around £3.7 million per year.^2^ In England, a postcode lottery plays a role in accessing the MT service. For example, about 10% of stroke patients receive a thrombectomy in London, compared with only 0–3% in other areas.^12^

At Hull Royal Infirmary, like many comprehensive stroke centres in the UK, we cannot provide a 24/7 MT service, mainly because of staffing issues. By implementing our project, we managed to establish the practice of early morning reimaging of the patients presenting out of hours who are eligible for MT. Utilising this approach granted more patients an access to this highly effective procedure. While the approach of reimaging patients may have already been used in other centres, this is the first description of its use in a formal structured way in centres where 24/7 MT service is not available.

We also managed to generalise this protocol to the district hospital in North Yorkshire and the Humber region. Our current policy states that patients will have the repeat imaging with CT/CTA/CTP by 6:30am in the district hospital and, if they are candidates for MT, they would be immediately transferred to the angio-suite in our hospital, allowing them to arrive by 8:00am. Following the procedure, patients are either repatriated directly to the referring hospital, or admitted to our hyperacute stroke unit if they are unstable or need observation for potential hemicraniectomy, which only takes place in our centre.

This QIP raises a debate regarding the proposed service extension: whether it is better to extend it to cover the weekends or to cover more hours on the weekdays. On one side, our data show that of the 81 patients originally reviewed, 14 patients (17%) underwent MT from November 2022 to March 2024, when the service was extended to cover from 4:00 pm to 8:00 pm on weekdays. While we did not collect the data for patients who presented at the weekend as they were not candidates for repeated imaging because of unavailability of MT on weekends, we can argue that extension of the MT service to cover the weekends, with application of our protocol, could provide the community with potential access to this treatment over the whole week.

Eventually, we must emphasise that this approach is not an alternative to extension of the MT service to cover 24/7. Also, we must accept the fact that a proportion of the patients that we rescan in the morning would not be eligible for this treatment, as shown in the results of the balancing measure.

## Conclusion

In stroke centres where a 24/7 MT service is not available, patients with LVO stroke who present in the non-operating hours could get access to this treatment by consideration of early morning rescanning, just before opening of the service. We think that our approach could be generalised to centres in the UK as a temporary solution until the service could be extended to cover 24/7.

## CRediT authorship contribution statement

**Shadi M Ramadan:** Writing – original draft, Methodology, Investigation, Formal analysis, Data curation, Conceptualization. **Bernard Esisi:** Writing – review & editing.

## Declaration of competing interest

The authors declare that they have no known competing financial interests or personal relationships that could have appeared to influence the work reported in this paper.

## Appendix Supplementary materials

Image, application 1
