# Supplementary material for: A quality improvement project on reimaging for provision of extended window mechanical thrombectomy when 24/7 service is not available
Source: Clin Med (Lond). 2025 Oct 9;25(6):100521. doi: 10.1016/j.clinme.2025.100521 (PMC12597272; doi:10.1016/j.clinme.2025.100521)
Supplement: Supplementary file 1 [file mmc1.docx]

Supplementary appendix

This appendix has been provided by the authors to give readers additional information about their work

Authors affiliations

Dr. Shadi Ramadan

Consultant stroke medicine

Hull University Teaching Hospitals NHS trust

Dr. Bernard Esisi

Consultant stroke medicine

Hull University Teaching Hospitals NHS trust

**Acknowledgement:**

The authors would like to thank Mrs Andrea Dennison (SSNAP coordinator) for providing the data for this work.

**Clinical Question and aim of the project:**

With the availability of the clinical evidence supporting the benefit of extended window mechanical thrombectomy and the unavailability of the 24-hour service in in our institution, the question was :how can we utilise the current evidence of extended window mechanical thrombectomy to provide this treatment to patients who present out of hours within the limited availability of the service?

We started our work with a baseline audit for the period from January 2021 to March 2022 to investigate the percentage of patients who managed to get access to mechanical thrombectomy when the service was not available.We found that only patients who presented in the early morning hours were considered for this treatment.

The details of this audit can be reviewed via the following link:

[A retrospective audit of out-of-hours mechanical thrombectomy of anterior circulation large vessel occlusion in a UK tertiary centre - PubMed](https://pubmed.ncbi.nlm.nih.gov/37775158/)

We designed a protocol at Hull Royal Infirmary to repeat CT head, CT angiogram and CT perfusion, to select patients who could still be candidate for mechanical thrombectomy when the service is open next morning. We distributed this protocol to the district hospitals (York Hospital and Scunthorpe Hospital) which refer the candidate patients to Hull Royal Infirmary.

**Protocol of reimaging:**


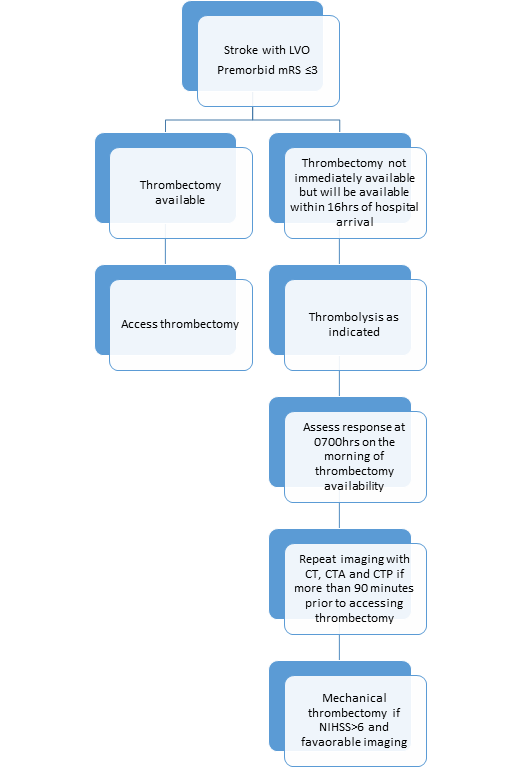


**Favourable imaging criteria is defined as:**

- Ischaemic core volume <70 ml.
- Mismatch ratio>1.8.
- Mismatch volume >15 ml.

**Patients from Hull Royal Infirmary (HRI):**

- Patients with NIHSS>6 will have reimaging at 7:00 am.
- Stroke consultant reviews the imaging.
- If the patient has favourable mismatch criteria, stroke consultant discusses the case with the interventional radiologist at 8:00 am to proceed with mechanical thrombectomy.
- Following the procedure, the patient is admitted to the hyperacute stroke unit (HASU) at HRI.

**Patients from district hospitals:**

- Patients with NIHSS >6 will have reimaging at 6:30 to 7:00 am.
- Stroke consultant at the district hospital reviews the images.
- If the patient has favourable mismatch criteria, stroke consultant at the district hospital informs the consultant stroke at HRI and arranges the transport. He will also contact the interventional radiologist at 8:00 am to inform him about the case. Being transferred.
- Following the procedure, the consultant at HRI will decide whether the patient can be repatriated to the district hospital or admitted to HASU at HRI for observation e.g. for potential hemicraniectomy.

**QIP design:**

**Eligibility**

Inclusion criteria (all these criteria must apply):

- Patients presented between 16:00 h and 08:00 h Monday to Thursday (MT available after 08:00 h the next working day).
- Patients presented to hospital at any time on a Sunday (MT would be available the following Monday).
- Onset of symptoms was <24 h from the next available time for MT.
- Premorbid pre-stroke Modified Rankin Score (mRS) ≤3.
- National Institutes of Health Stroke Scale (NIHSS) ≥6.
- Evidence of anterior circulation LVO on imaging.

**Exclusion criteria:**

Premorbid mRS >3.

NIHSS <6.

Post-lysis NIHSS <6 (if the patient had been thrombolysed).

No evidence of LVO on imaging/low mismatch on perfusion.

Onset of symptoms >24 h from the next available time for MT.

Patients presented on Saturday or after 16:00 h on Friday (because MT service would not be available within the following 24 h).

**Data collection:**

**591 patients were admitted with ischaemic stroke during the period of the QIP from August 2022 to June 2024. Of those, 81 patients had large vessel occlusion (LVO).**

**42 patients were excluded as shown in the table**

| Cause of exclusion | Number of patients |
| --- | --- |
| Presentation within the extended MT service From November 2022 to March 2024) | 14 |
| Presentation in the early morning hours | 11 |
| NIHSS on presentation <6 | 6 |
| Post lysis NIHSS <6 | 3 |
| Posterior circulation stroke | 3 |
| Premorbid MRS>3 | 3 |
| Frailty | 1 |
| Unfavourable perfusion criteria on the first scan | 1 |

**Analysis:**

Data was analysed retrospectively on a 4 monthly basis from August 2022 to June 2024 using the SSNAP record at Hull Royal infirmary.

| **Category** | Value |
| --- | --- |
| **Total Patients** | 39 |
| **Gender** |  |
| Men | 24 (67%) |
| Women | 15 (33%) |
| **Mean Age** | 70 years |
| **Patients ≥80 years** | 12 (31%) |
| **Source** |  |
| Comprehensive stroke centre | 33 patients admitted directly to Hull Royal infirmary |
| District hospitals | 4 patients referred from York and 2 patients fromScunthorpe Hospitals |
| **Thrombolysis** |  |
| - yes | 26 patients |
| - No | 13 patients |
| **Initial NIHSS Score** |  |
| – 6 to 12 | 9 patients (23%) |
| – 13 to 20 | 20 patients (51%) |
| – >20 | 10 patients (26%) |
| **Median NIHSS** |  |
| Before thrombolysis | 18 |
| After thrombolysis | 10 |

**Data of patients included in the QIP:**

| source | Age | Gendre | MRS | NIHSS | Post-lysis NIHSS | Repeat scan done? | MT done | MT outcome (TICI) |
| --- | --- | --- | --- | --- | --- | --- | --- | --- |
| HRI | 64 | M | 1 | 24 | Not lysed | No | No |  |
| HRI | 41 | F | 0 | 20 | 8 | Yes | No |  |
| SGH | 65 | M | 0 | 23 | 10 | Yes | Yes | 2b |
| HRI | 74 | F | 2 | 14 | 6 | No | No |  |
| HRI | 60 | M | 1 | 6 | 4 | Yes | No |  |
| HRI | 65 | M | 1 | 12 | 9 | Yes | Yes | 2b |
| HRI | 70 | M | 0 | 24 | 18 | Yes | No |  |
| HRI | 64 | M | 2 | 14 | Not lysed | No | No |  |
| HRI | 64 | M | 1 | 15 | 6 | Yes | No |  |
| HRI | 47 | M | 0 | 28 | 6 | Yes | No |  |
| HRI | 90 | F | 2 | 16 | Not lysed | Yes | Yes | 3 |
| HRI | 85 | M | 2 | 11 | 8 | yes | No |  |
| HRI | 81 | F | 0 | 25 | 12 | yes | No |  |
| HRI | 76 | M | 3 | 20 | 16 | Yes | yes | 2C |
| HRI | 76 | F | 1 | 18 | Not lysed | Yes | yes | 2C |
| YH | 35 | F | 1 | 13 | Not lysed | Yes | Yes | 1 |
| HRI | 84 | M | 1 | 10 | 6 | yes | Yes | 3 |
| SGH | 65 | M | 0 | 11 | 11 | yes | Yes | 3 |
| YH | 67 | F | 0 | 18 | 17 | Yes | Yes | 3 |
| HRI | 55 | M | 0 | 15 | 15 | Yes | Yes | 2C |
| HRI | 57 | M | 0 | 6 | 3 | Yes | No |  |
| HRI | 86 | F | 2 | 17 | Not lysed | yes | Yes | 0 |
| HRI | 87 | F | 2 | 24 | 24 | yes | No |  |
| HRI | 74 | M | 1 | 6 | 4 | Yes | yes | 3 |
| HRI | 74 | F | 0 | 18 | 13 | Yes | yes | 2c |
| HRI | 88 | M | 2 | 24 | 15 | Yes | yes | 3 |
| HRI | 88 | F | 1 | 23 | Not lysed | yes | yes | 3 |
| HRI | 58 | F | 1 | 15 | Not lysed | yes | No |  |
| HRI | 82 | M | 1 | 13 | 6 | Yes | No |  |
| HRI | 89 | F | 2 | 19 | 16 | Yes | No |  |
| HRI | 83 | M | 0 | 7 | 11 | Yes | No |  |
| HRI | 71 | M | 1 | 19 | 3 | yes | Yes | 3 |
| HRI | 50 | M | 1 | 17 | 6 | yes | No |  |
| HRI | 52 | F | 0 | 19 | 13 | yes | yes | 3 |
| YH | 40 | F | 1 | 8 | 8 | yes | yes | 3 |
| YH | 59 | M | 0 | 20 | 5 | yes | yes | 2C |
| HRI | 70 | M | 1 | 20 | Not lysed | yes | yes | 3 |
| HRI | 88 | M | 2 | 26 | 14 | yes | yes | 3 |
| HRI | 66 | M | 1 | 21 | 11 | yes | yes | 3 |

Abbreviations:

MRS: modified Rankin score., NIHSS: National institute of health scale, MT: mechanical thrombectomy,TICI: thrombolysis in cerebral infarction score, HRI: Hull Royal infirmary, SGH: Scunthorpe General hospital, YH: York hospital,

**Causes why patients were not accessed MT after repeated imaging.**

| Reason why a patient was not considered for MT | Number |
| --- | --- |
| Reduced mismatch volume in the repeated perfusion scan | 5 |
| Re-canalisation was achieved in the repeated CT angiogram scan after thrombolysis | 7 |
| Discretion of the interventional radiologist (Those two patients’ ages were 87 and 89 years and were considered for repeated imaging by the on-call stroke consultant at night but, they were rejected after discussion with the interventional radiologist in the morning. | 2 |

**Data of patients who had mechanical thrombectomy when the extended service was available from 16:00 to 20:00 (excluded from the QIP).**

| source | Age | Gendre | MRS | NIHSS | Post-lysis NIHSS | MT outcome (TICI) |
| --- | --- | --- | --- | --- | --- | --- |
| HRI | 59 | M | 0 | 17 | 10 | 0 |
| HRI | 78 | F | 3 | 24 | Not lysed | 3 |
| SGH | 70 | M | 2 | 12 | Not lysed | 3 |
| HRI | 83 | M | 0 | 6 | Not lysed | 2C |
| HRI | 88 | F | 0 | 10 | Not lysed | 3 |
| HRI | 94 | M | 4 | 3 | Not lysed | 3 |
| HRI | 78 | F | 1 | 18 | Not lysed | 2C |
| HRI | 76 | F | 1 | 20 | 5 | 3 |
| HRI | 86 | F | 0 | 16 | Not lysed | 2C |
| HRI | 79 | M | 3 | 15 | Not lysed | 3 |
| HRI | 88 | F | 1 | 14 | Not lysed | 2b |
| HRI | 62 | F | 1 | 23 | 10 | 0 |
| HRI | 60 | M | 1 | 8 | 0 | 2b |
| HRI | 61 | M | 1 | 14 | 3 | Not recorded |
